# Experimental Investigation of Grinding Force and Material Removal Mechanism of Laser-Structured Zirconia Ceramics

**DOI:** 10.3390/mi13050710

**Published:** 2022-04-30

**Authors:** Jingzhu Pang, Xia Ji, Yan Niu, Shaojun Chen

**Affiliations:** 1 College of Mechanical Engineering, Donghua University, Shanghai 201620, China; jixia@dhu.edu.cn (X.J.); 2211008@mail.dhu.edu.cn (Y.N.); 2 Shanghai Collaborative Innovation Center of High Performance Fibers and Composites, Donghua University, Shanghai 201620, China; 3 YoanTion Industrial Inc., Ltd., Shaoxing 312599, China; junshaochen@163.com

**Keywords:** laser-assisted grinding, grinding force, material removal mechanism, microcracks

## Abstract

Zirconia is a high demanded structural ceramic with desirable mechanical, thermal, and chemical properties. Poor surface integrity and limited material removal rate caused by high cutting force and wheel wear are the main problems in ceramic grinding. In order to reduce the grinding force and enhance the removal rate in grinding, zirconia ceramics are firstly ablated by laser and then be grinded. A nanosecond laser is used to ablate the surface of zirconia ceramic, the laser-ablated structures with micro pits and thermal microcracks are generated. With the input of subsequent grinding, the machinability of zirconia ceramic workpiece with laser-ablated structures changes. Grinding experiments are conducted to study the grinding force and the material remove of laser-structured zirconia ceramic. Results show that the grinding forces in tangential and normal direction are significantly reduced. Compared to the grinding surface without laser-structured, a damage-free grinding surface is obtained by laser assistance.

## 1. Introduction

Owing to the superior combination of physical and mechanical properties, advanced ceramic materials such as zirconia, silicon nitride, and alumina are widely used in precision bearing of high-grade machines, such as wind turbines, high-grade CNC machine tools, and heat settings. Laser assisted machining (LAM) mainly aims at improving the machining efficiency and cutting performance of difficult-to-machining materials, such as Al_2_O_3_ [1], Si_3_N_4_ [2] and ZrO_2_ [3]. Based on the local softening mechanism, most LAM use laser to instantly heat and soften the local area of ceramic surface, and then carry out tool cutting, to obtain continuous chips and reduce the cutting force [4].

If the temperature or thermal stress of the material caused by the laser reaches the threshold, irreversible thermal damage, including cleavage, melting and vaporization will be generated [5]. The microcracks induced by thermal stress on the surface and inside will result in the reduction in material strength. Sun et al. [6] pointed out that by preheating the material before cutting, the yield strength of the material can be reduced, so that the ceramic machining mechanism can be transformed from brittle to ductile. Marinescu et al. [7] conducted laser preheating grinding on four ceramic materials, and found that laser preheating grinding can not only reduce the hardness of ceramics and improve the remove rate, but also avoid grinding cracks.

Tsai et al. [8] used laser thermal stress to realize laser assisted milling of pits on the surface of ceramic samples, pointed out that the tensile stress perpendicular to the laser scanning path is the largest, and controlled the growth of cracks. Wang et al. [9] used heat to improve the mechanical properties of quartz glass in the grinding and realized the efficient ductile grinding of quartz glass. Azarhoushang et al. [10] identified that the reduced specific grinding energy through laser structuring of workpiece is mainly due to the induced lateral cracks. Xu et al. [11] also showed that laser irradiation on zirconia ceramics prior to the grinding process led to development of thermal damages on the surface of the workpiece, which contributed to the decrease in the specific grinding energy. Ma et al. [12] built a grinding force predictive model for the LAG process that reveals the mechanism for the reduction in grinding force during LAG on zirconia ceramics. Li et al. [13] revealed the structural changes and hardness decrease enhanced the probability of plastic removal in LAG and obtained better surface integrity. Zhang et al. [3] presented a theoretical grinding force model by taking into account the three grinding stages in laser macro-micro-structured grinding (LMMSG). The model may be available to predict the grinding force of zirconia ceramics under the LMMSG condition.

Kumar et al. [14], Zhang et al. [15] proposed that laser irradiations should be carried out before processing silicon nitride ceramics to induce cracks and microcracks on the surface, reduce the strength of the material and improve the machinability. Pratap et al. [16] investigated the material removal mechanism and analyzed the damage occurrence for parallel as well as intersecting micro grinding. Rao et al. [17] revealed that adhesion and pullout of diamond grits in laser-assisted grinding were remarkable difference from those in conventional grinding due to the thermal softening of both RB-SiC specimen and bond material of the grinding wheel. Kadivar et al. [18] showed that the laser-cut depth could be predicted and choosing optimal laser parameters is possible to achieve a certain laser-cut depth. Yang et al. [19] proposed a heat flux theoretical model based on the dynamic grinding force of hard-brittle bone ductile micro-grinding to solve this bottleneck problem. 

In laser-assisted grinding (LAG) process, laser thermal effect and grinding effect are successively loaded on workpiece surface, the grinding force and material removal mechanism will be different from that of grinding. However, few research has been conducted on the benefits of applying LAG on zirconia. In this paper, a LAG process is studied for applying a nanosecond laser ablation before grinding of zirconia. Zirconia workpieces will be laser-structured as the first step, then grinding experiments are carried out to study the effects of laser-generated microstructures and their corresponding parameters on grinding forces and material removal mechanism.

## 2. Experimental Setup and Procedures

### 2.1. Laser Pretreatment 

**Figure 1:**
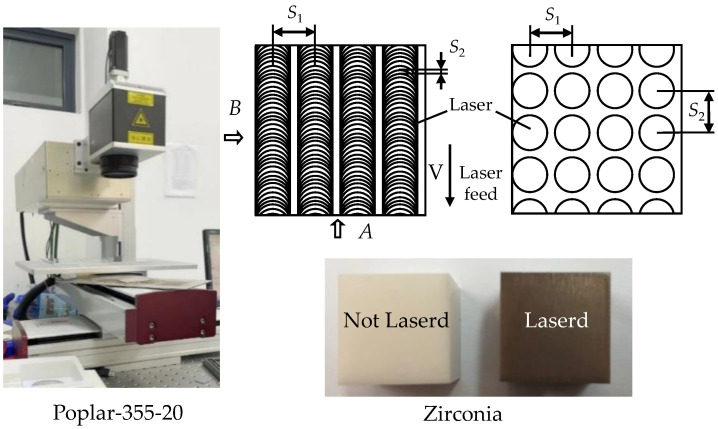
Experimental setup of laser pretreatment.

The laser conditioning setup is shown in Figure 1. A nanosecond laser (poplar-355-20) is used to ablate the surface of zirconia workpiece. The maximum laser power is 20 W with a wavelength of 355 nm. Zirconia is selected as the workpiece material, of which the size is 15 mm × 15 mm × 15 mm. The main parameters of the laser are shown in Table 1. Four parameters are mainly considered in laser ablation process, such as laser frequency, laser line span, laser scan speed, and irradiation times.

The laser moves vertically and focuses the beam on the workpiece surface. The workbench is equipped with a cross slide so that the laser can follow a specified path on the workpiece surface. Direction *A* is the scanning direction of the laser, and direction *B* is the laser line span. Span *S*_1_ and *S*_2_ directly affect the overlap of two adjacent laser spots and have a direct effect on the laser processing efficiency and quality. The parameters of Poplar-355-20 are shown in Table 1.

### 2.2. Grinding Force Monitoring

**Figure 2:**
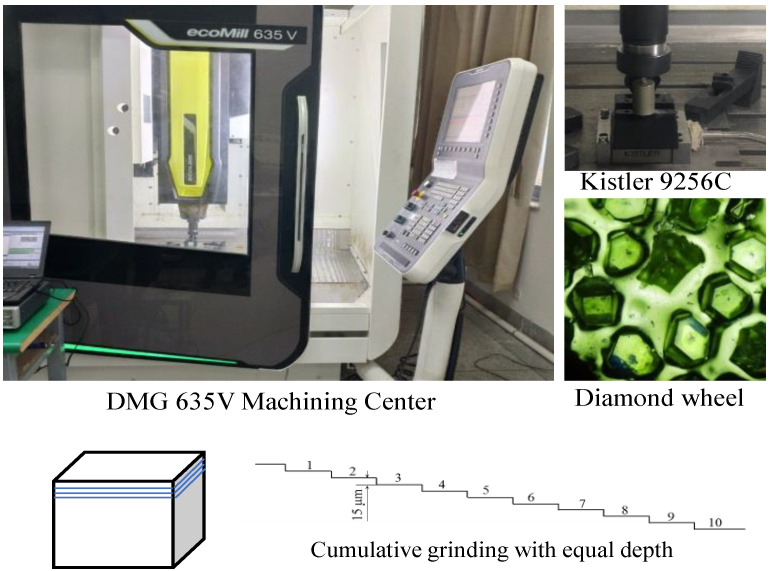
Experimental setup for grinding force monitoring.

Grinding force experiments on the zirconia samples were carried out on a CNC machining center (ecoMill 635 V, DMG MORI, Shanghai, China) equipped with a dynamometer Kistler 9256 C, as shown in Figure 2. Normal and tangential grinding force components were measured during the grinding process by this dynamometer. The dynamometer is equipped with an amplifier Kistler 5080 A. DAQ system (LMS) was used for data acquisition (sampling frequency 6400 Hz). The diameter of electroplated diamond wheel (HS-JGPC10) is Φ10 mm, and the size of abrasive grain is about 120 μm. Grinding parameters are shown in Table 2.

In up grinding process, the grinding wheel rotates in clockwise and the workpiece moves in directions X and Y. The workpiece moves from the left side of the grinding wheel to its right side, and the grinding force is recorded. When the material is removed by the layer thickness of 15 μm, the workpiece will return to the left, and the grinding for the second layer with the depth of 15 μm will be carried out with the same grinding parameters. In order to carry out grinding experiments with identical conditions, the grinding wheel is replaced in time to retain the same micromorphology.

### 2.3. High-Speed Grinding

**Figure 3:**
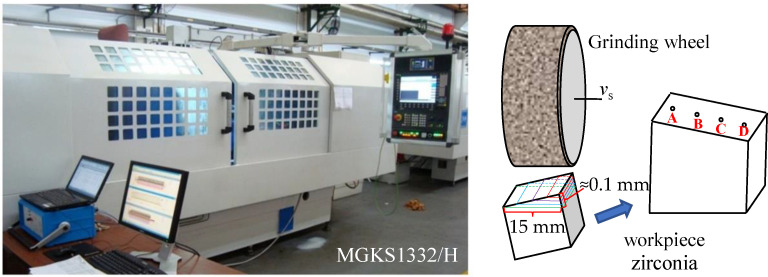
Experimental setup for high-speed grinding.

High-speed grinding of zirconia ceramics with laser ablation was conducted on a high-speed grinding machine (MGKS1332/H, SMTW, Shanghai, China) as shown in Figure 3. A vitrified bond diamond wheel (D91 V + 2046J1SC C150E, Φ400 × 22 × 203.5) was employed. The size of the diamond grit was approximately 91 μm. The diameter of the wheel was 400 mm, and the width was 22 mm. Before up grinding, the wheel was balanced using a dynamic balancing instrument (SB-4500, SMIT, Portland, OR, USA). Zirconia workpiece is slightly inclined and installed on the fixture (the tilted angle is shown in Figure 3). Wheel speed *v*_s_ = 60 m/s, workpiece speed *v*_w_ = 0.1 m/s, and depth of cut *a*_p_ = 15 μm are used for grinding in order to obtain surfaces with different grinding removal depths under the same process conditions.

## 3. Results and Discussion

### 3.1. Laser Ablated Surface

The laser pretreatment parameters for zirconia ceramic workpieces are listed in Table 3. Laser spot diameter is about 0.05 mm. The laser line span *S*_1_ is variable, which is 40 μm, 60 μm, 80 μm and 100 μm, respectively. The scan speed can be determined by the distance *S*_2_ and the pulse frequency. If the laser pulse frequency is 49 Hz, *S*_2_ is about 40 μm, the scan speed is about 1960 mm/min. The laser power is changed by adjusting the pulse frequency, and when the pulse frequency is 50 Hz, the laser power equals to 20 W. Three samples are processed for each laser parameters.

**Figure 4:**
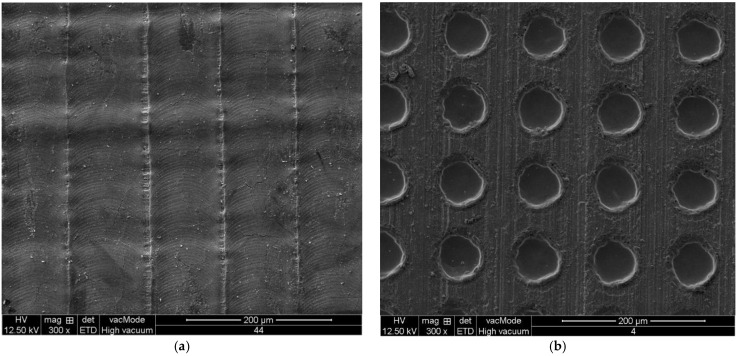
Two laser-structured workpieces with different laser parameters (*f* = 49 kHz, *V* = 4900 mm/min, *S*_1_ = 100 μm). (**a**) *N* = 1; (**b**) *N* = 40.

The surface microtopography was examined by an environment scanning electron microscope (QUANTA 250, FEI, Brno, Czech Republic). Two laser-structured workpieces with different laser parameters are illustrated in Figure 4. From Figure 4a, it is found that when the laser scanning speed is 4900 mm/min, the lasered structure of group pits are difficult to see after one scan. However, when the laser ablates 40 times at the same position of the workpiece surface, the lasered structure of group pits is clear to see, as shown in Figure 4b.

**Figure 5:**
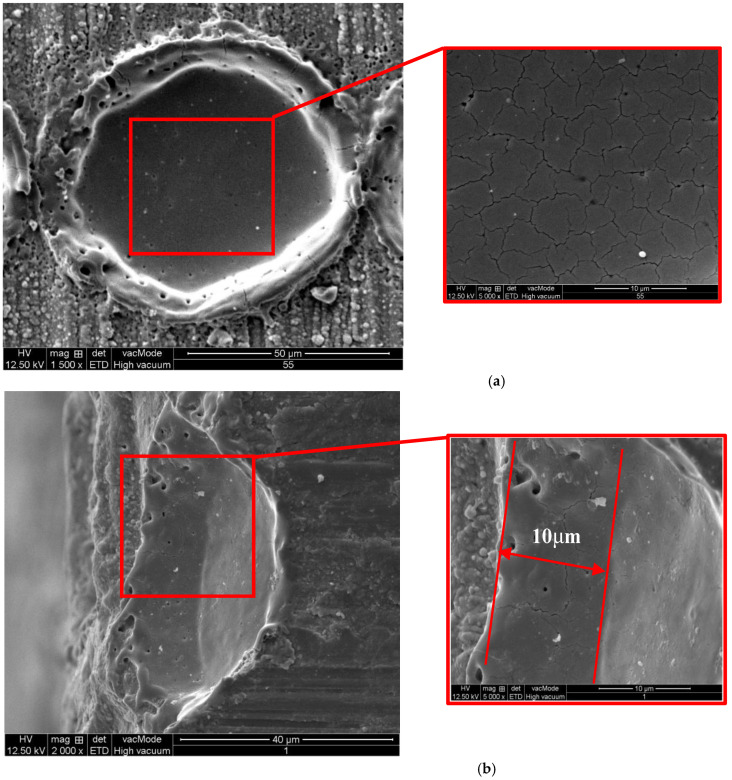
SEM microtopography of a laser pit on zirconia workpiece (*f* = 49 kHz, *V* = 100 mm/min, *S*_1_ = 100 μm, *N* = 1). (**a**) Top View, (**b**) Section view.

Figure 5 shows the SEM microtopography of the zirconia workpiece. From Figure 5a, we can see that the diameter of the laser spot is about 50 microns. The surface is melted by the laser, which generates heat-affected zone and thermal microcracks. The material around the pit is melted and raised with microcracks at the protrusion. As the temperature of the material increases rapidly, the material expands and compressive stress is generated. When the heat source leaves, the temperature drops quickly and the material shrinks, resulting in the tensile stress which would lead to crack initiation, material fracture and rapid reduction in stress.

From Figure 5b, it is seen that the bottom is relatively flat with lateral cracks on it. The top diameter is about 50 μm, and the depth is about 10 μm. There are no median cracks on subsurface below this pit. In Figure 5b, it is found that the propagation of intergranular microcracks leads to the microcrack network in the laser ablated area. These microcracks caused by laser is initiated by the defects on the ceramic surface and expanded by the thermal stress. 

**Figure 6:**
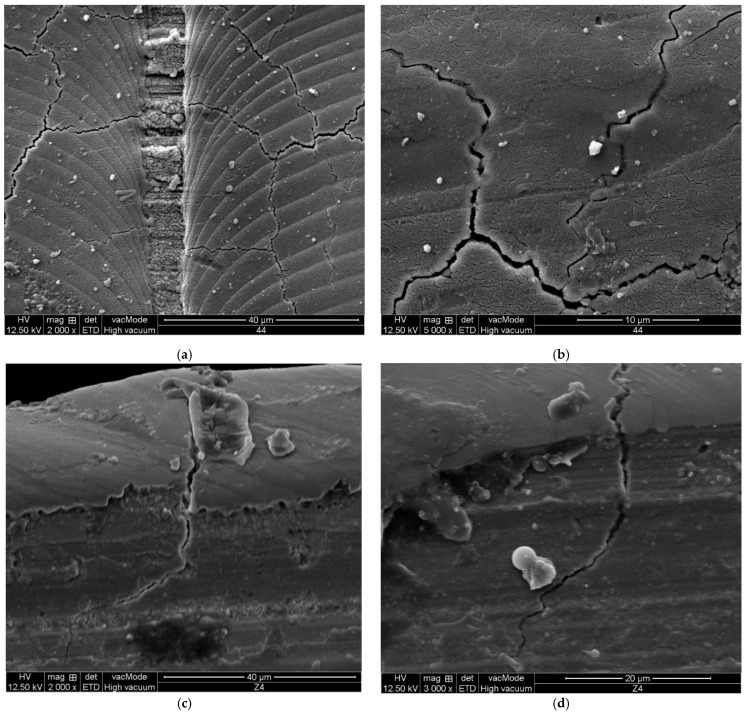
SEM microtopography of cracks on lasered zirconia workpiece (*f* = 49 kHz, *V* = 100 mm/min, *S*_1_ = 100 μm, *N* = 1). (**a**) Lateral cracks. (**b**) Lateral cracks (Enlarged). (**c**) Median crack (Position 1). (**d**) Median crack (Position 2).

As the laser moves slowly, the laser spots gradually overlapped. The total laser energy on the zirconia surface with scan speed of 100 mm/min by once (as shown in Figure 6) and with scan speed of 3920 mm/min by 40 times (as shown in Figure 5) are approximately equal, while the surface morphology is completely different. The diameter of the laminated arc formed by the laser on the workpiece surface is much larger than the spot diameter. The latter laser reprocesses some areas of the surface affected by the previous laser. The width of a single microcrack on the workpiece surface in Figure 6a,b is greater than that in Figure 5, which also shows that the depth of the microcracks will be relatively deeper. By comparing Figure 6 with Figure 5, it can be noticed that not only lateral cracks, but also median cracks are generated in slow speed scanning. In Figure 6c,d, there are median cracks, which extends to the subsurface of the material by 20~30 μm. The existence of microcracks will reduce the material strength, which is helpful for subsequent grinding.

### 3.2. Grinding Force

**Figure 7:**
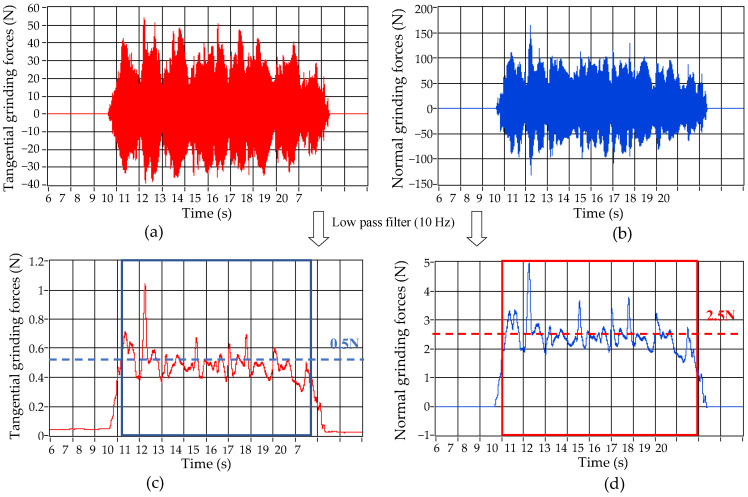
Grinding force (No irradiation, *v*_s_ = 3000 RPM, *v*_w_ = 0.1 m/min, *a*_p_ =15 μm). (**a**) Original signal of Tangential force. (**b**) Original signal of normal force. (**c**) Filtered signal tangential force. (**d**) Filtered signal of normal force.

Figure 7a,b show the grinding force of the workpiece without laser irradiation. The grinding wheel rotates at 3000 rpm, the workpiece approaches the wheel at a speed of 0.1 m/s, and the grinding depth is 15 μm. As the workpiece contacts with the wheel, the amplitude of the signal increases rapidly. As the workpiece leaves the grinding wheel, the amplitude of the grinding force quickly returns to zero.

The original signal is low-pass filtered, and the low-pass frequency is 10 Hz. The tangential grinding force and normal grinding force are shown in Figure 7c,d, respectively. Intercept the signals in the stable stage of grinding, which are shown in the box in Figure 7c,d. Remove the gross errors, and take their mean values as the results of grinding force. The average value of filtered normal grinding force is about 2.5 N, as the dotted line in Figure 7c,d.

**Figure 8:**
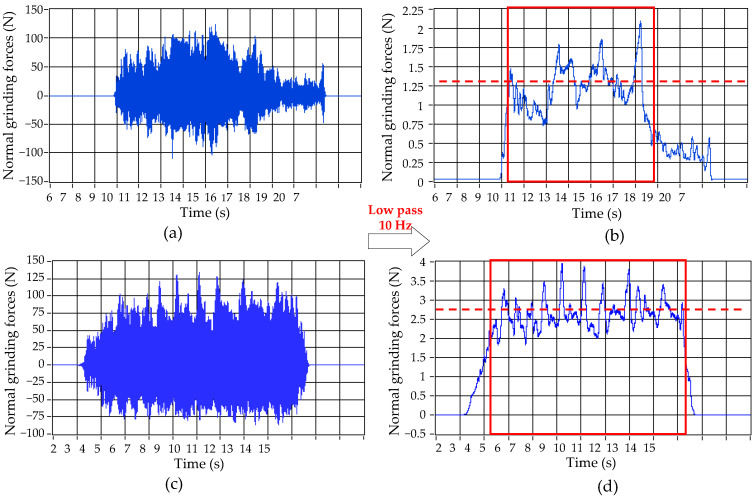
Normal grinding force (*k* = 20 kHz, *V* = 1600 mm/min, *S*_1_ = 80 μm, *N* = 40). (**a**) Original signal (Layer: 0–15 μm). (**b**) Filtered signal (Layer: 0–15 μm). (**c**) Original signal (Layer: 75–90 μm). (**d**) Filtered signal (Layer: 75–90 μm).

The normal grinding force signals of the layer with 0–15 μm and 75–90 μm are illustrated in Figure 8. From Figure 8a,b, it is found that for the 0–15 μm layer, the filtered signals of normal grinding force fluctuate greatly, which RMS increases significantly. It is also found that there are micro pits, protrusions and microcracks on the lasered surface, and laser ablation changes the mechanical properties along the workpiece depth. The average amplitude of the filtered signal decreases significantly compared with the value in Figure 7d. From Figure 8c,d, it is found that when the laser ablation is all removed, the normal grinding force returns to about 2.5 N.

**Figure 9:**
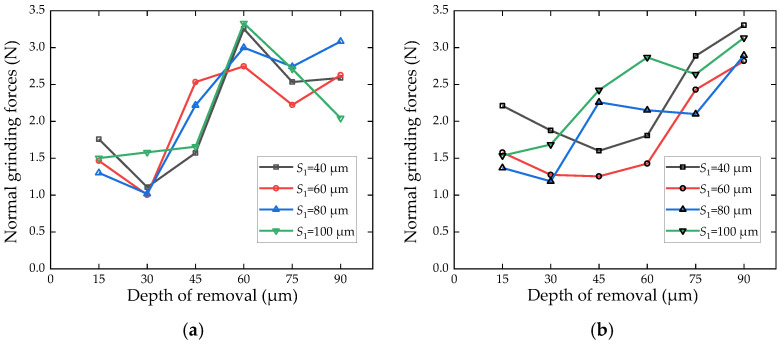
The relationship between the normal grinding force the total material removal thickness (*N* = 40). (**a**) *k* = 49 kHz. (**b**) *k* = 20 kHz.

The relationship between the normal grinding force and the depth of removal is shown in Figure 9. The data in Figure 9 are the average values calculated by the grinding forces of the workpiece with the same laser parameters. It shows that the normal grinding force increases with the increasing of the grinding depths. When the distance is far away from the workpiece surface, the effect of laser irradiation is not significant. The reason for the rise of grinding force is that the unaffected ceramic materials gradually join the grinding process. The grinding resistance of materials without microcracks is greater than that of materials with laser irradiated microcracks. As the laser-affected layer generated by laser irradiation is removed completely, the grinding force stabilizes to an asymptotic value shown in Figure 7. It was noted that the maximum reduction in grinding force is more than 40%. However, the maximum falling amplitude is not at 0–15 μm layer thickness, but about at 75–90 μm layer thickness. At first, the laser ablation layer on the workpiece surface is removed. As the top layer is removed, there are the intersections of median cracks and lateral cracks on the left surface, which will lead to the decrease in its hardness. As the abrasive grains land on the cracked surface, the cracked surface is further broken and separated from the surface under the action of mechanical stress.

From the comparison between Figure 9a,b, it is found that the overall decrease in grinding force is significant under high frequency, and its laser energy is much larger. It is also found that the smaller *S*_1_ is, the stronger the material is affected by the laser and the deeper the ablation influence layer obtains. The trend of normal grinding force of four cases is relatively consistent under high frequency. Under low frequency, the curves of the laser line span 80 μm and 100 μm rise earlier than that of 40 μm and 60 μm, because the laser ablation influence layer of 40 μm and 60 μm is greater than that of 80 μm and 100 μm.

**Figure 10:**
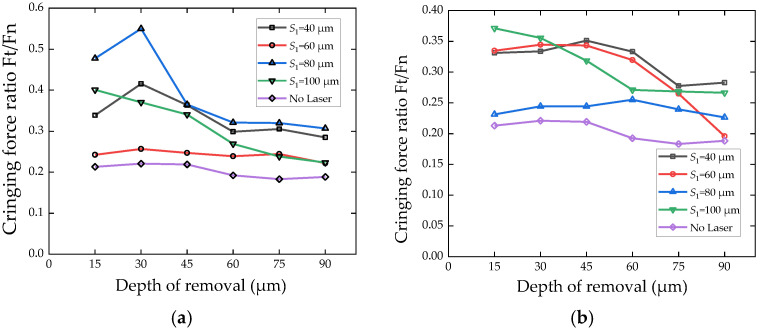
The relationship between the normal grinding force the total material removal thickness (*N* = 40). (**a**) *k* = 49 kHz. (**b**) *k* = 20 kHz.

Grinding force ratio (*F*t/*F*_N_) is an index reflecting material brittleness in traditional hard and brittle material grinding. Large brittleness leads to small force ratio. Figure 10 shows that laser ablation pretreatment can improve grinding force ratio. The surface hardness of zirconia ceramics decreases due to laser ablation, and the normal load required for abrasive grains to invade the material decreases. In the initial stage of grinding, there are many brittle fractures in material removal, which also shows that laser irradiation increases the brittleness of ceramics. The influence depth of laser ablation on the grindability of the workpiece exceeds the depth of the ablation influence layer observed in Figure 5 and Figure 6. Compared with Figure 10a,b, it is found that high laser energy leads to high grinding force ratio during workpiece surface removal.

### 3.3. Material Removal in Low-Speed Grinding

**Figure 11:**
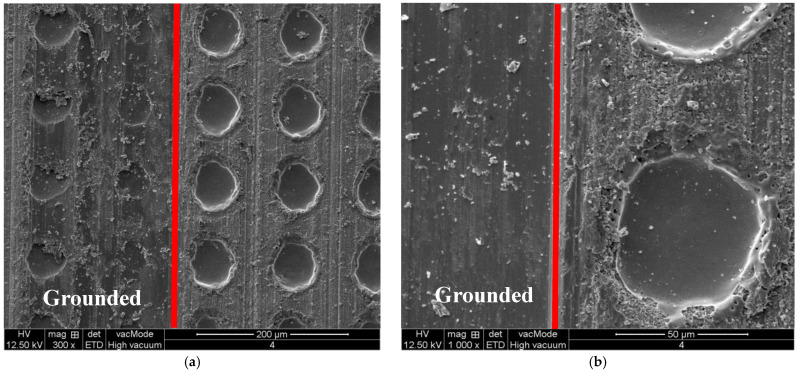
SEM microtopography of the lasered surface which is half grounded. (**a**) Partially grounded. (**b**) All grounded.

Figure 11 shows the SEM microtopography of the surface of a workpiece, of which the left side is the grounded area. From Figure 11a, it is found that the remelting protrusion around the original lasered pits are partially removed, and micro grinding chips are left on the surface. In grinding, when the abrasive grains ground material surface, the normal and tangential forces lead to the generation of median cracks and lateral cracks on the material surface and sub surface, respectively. From these SEM microtopography, It can be found that there are many interconnected lateral microcracks on the surface of the pits. The stress generated by the contact between abrasive grains and workpiece will lead to the generation of microcracks. However, propagation of these microcracks caused by grinding will be interrupted by the microcracks already caused by laser. The material surrounded by lateral cracks is broken into smaller fragments. As shown in Figure 11b, the remelting protrusion around the original lasered pits are all removed by grinding, and the damage of median and lateral cracks caused by the previous laser ablation does not affect the integrity of the newly created surface.

**Figure 12:**
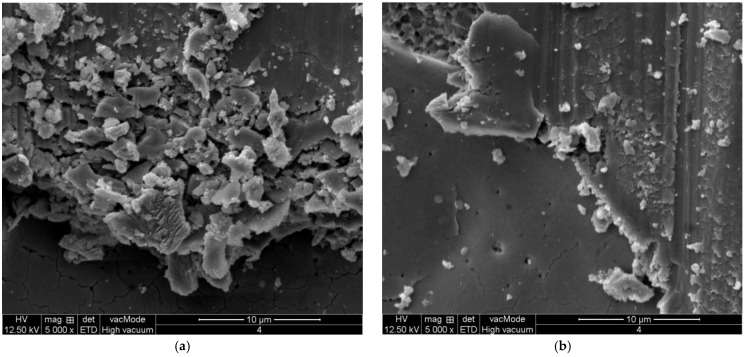
Grinding chips and plastic scratches near lasered pits. (**a**) Chips. (**b**) Plastic scratches.

The frequent impacts between grain and workpiece at laser pit will result in powerful mechanical shocks. As shown in Figure 12a, the size of grinding chips is mostly less than 10 μm in size, which is less than the grid size formed by the microcracks at the bottom of lasered pits or around the pits before grinding. Therefore, the new and smaller microcracks are produced by grinding mechanical stress, which improve the further crushing of the material surface. Some broken pits have not yet fallen off the surface. As the grinding process continues, these pits will be further crushed or separated from the surface, as shown in Figure 12b. With the further removal of materials, the depth of lateral cracks remaining on the newly formed grinding surface decreases.

### 3.4. Material Removal in High-Speed Grinding

**Figure 13:**
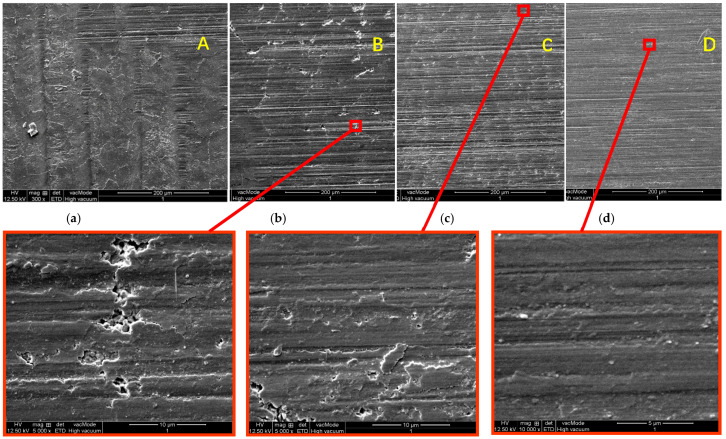
Micromorphology of the workpiece produced by high-speed grinding. (**a**) Surface *A*. (**b**) Surface *B*. (**c**) Surface *C*. (**d**) Surface *D*.

Figure 13 shows the micromorphology of the workpiece in Figure 3 which is grounded by MGKS1332/H. Figure 13a–d show the surfaces at different positions of the same workpiece after grinding, in which the material removal of surface *A* is the smallest and that of surface *D* is the largest. The layers irradiated by laser on Surface *D* are all removed. From Figure 13, it is found that there are some pits and microcracks on Surface *B* and *C*, while no microcracks are found on Surface *D*.

It is also found that there are obvious grids of laser irradiated microcracks on Surface B. In the enlarged picture of Surface *B*, material fractures and the scratches of abrasive grains can be found. Combined with the previous analysis results of grinding force, the removal of laser ablated layer should be dominated by brittle removal. As the grinding wheel speed increases, the maximum undeformed chip thickness decreases and the material is more easily removed by ploughing. The resistance of the material to crack generation and propagation increases. The speed of the grinding wheel used here is 60 m/s, and the corresponding surface characteristics show that the grinding in the laser ablated layer is in a mixed material removal mode which combined brittle material removal with ductile material removal

On Surface C, the laser-structured and influenced layer is further removed, but the grid traces left by lateral cracks still can be seen on the surface. Obvious grinding marks can be seen on Surface D, on which there is no more visible lateral cracks. There are small and shallow defects and irregularities which are formed by abrasive grains. They are typical features of ductile material removal.

The above results show that the surface cracks introduced by laser ablation will improve the grindability and reduce the grinding force of zirconia ceramic, and the cracks will not expand into the matrix after the influenced layer of laser is completely removed. The surface quality produced by laser-structured grinding is consistent with that of grinding.

## 4. Conclusions

In this paper, different laser-structured workpieces are made to investigate the grinding force and material removal mechanism of laser-structured zirconia ceramics. The grinding force of the workpiece is experimentally studied, and the maximum reduction in grinding force is more than 40% for the laser ablated surface. The microcracks caused by the laser not only prevent the further propagation of the median cracks generated by grinding, but also improve the material removal rate. The grinding experiments show that low-speed grinding of laser ablation affected layer presents mainly brittle removal, while with the increase in grinding wheel speed, high-speed grinding presents mainly ductile removal.

## Figures and Tables

**Table 1:** Parameters of Nanosecond UV Laser system.

Characteristics	Symbol	Unit	Values
Average power	*P*_ap_	[W]	0.02–20.61
Wavelength	*λ*	nm	355
Maximum pulse power	*L*_p_	kW	8
Pulse energy	*J*_e_	mJ	0.4
Pulse duration	*T*_D_	ns	50
Pulse frequency	*f*	[kHz]	20~50
Scan speed	*V*	[mm/s]	1~5000

**Table 2:** Grinding parameters on DMG 635 V.

Wheel Speed *v*_s_ [m/s]	Workpiece Speed *v*_w_ [m/s]	Cutting Depth *a*_p_ [μm]
1.57	0.0017	15

**Table 3:** Laser processing parameters.

Characteristics	Symbol	Unit	Values
Pulse frequency	*f*	[kHz]	20, 49
Scanning speed	*V*	mm/min	Determined by *f* and *S*_2_
Number of scans	*N*	/	1, 20, 40
Laser line span	*S*_1_	μm	40, 60, 80, 100

## Data Availability

Not applicable.
